# *Elizabethkingia anophelis* outer membrane vesicles as a novel vaccine candidate against infection: insights into immune response and potential for passive immunity

**DOI:** 10.1128/msphere.00400-23

**Published:** 2023-11-28

**Authors:** Ya-Sung Yang, Hung-Jui Chen, Xiao-Chun Chen, Hung-Jen Tang, Fang-Ju Chang, Yun-Ling Huang, Yu-Ling Pan, Dinesh Kumar Kesavan, Huan-Yuan Chen, Hung-Sheng Shang, Shu-Chen Kuo, Te-Li Chen, Ming-Hsien Chiang

**Affiliations:** 1 Department of Internal Medicine, Division of Infectious Diseases and Tropical Medicine, Tri-Service General Hospital, National Defense Medical Center, Taipei, Taiwan; 2 Department of Infectious Diseases, Chi Mei Medical Center, Tainan, Taiwan; 3 Department and Graduate institute of Microbiology and Immunology, National Defense Medical Center, Taipei, Taiwan; 4 Department and Graduate Institute of Biology and Anatomy, National Defense Medical Center, Taipei, Taiwan; 5 School of Material Science, Nanyang Technological University, Singapore, Singapore; 6 Inflammation Core Facility, Institute of Biomedical Sciences, Academia Sinica, Taipei, Taiwan; 7 Department of Pathology, Division of Clinical Pathology, Tri-Service General Hospital, National Defense Medical Center, Taipei, Taiwan; 8 National Institute of Infectious Diseases and Vaccinology, National Health Research Institutes, Zhunan, Taiwan; 9 Graduate Institute of Life Sciences, National Defense Medical Center, Taipei, Taiwan; U.S. Food and Drug Administration, Silver Spring, Maryland, USA

**Keywords:** *Elizabethkingia anophelis*, outer membrane vesicle, immunization, passive immunity, vaccines

## Abstract

The emerging pathogen *Elizabethkingia anophelis* poses severe threats to public health and has caused several outbreaks. The efficacy of antimicrobials is limited due to the pathogen’s innate multi-drug resistance to most antibiotics. Hence, novel approaches to treat *E. anophelis* infections are urgently needed. This study evaluated the immunogenic and protective effects of the imipenem-induced outer membrane vesicles (iOMVs) of *E. anophelis* as a vaccine. Mice immunized with iOMVs were completely protected during lethal-dose challenges. Passive immunization with hyperimmune sera and splenocytes conferred protection against lethal pneumonia. iOMVs also induced Th1-, Th2-, and Th17-driven immune responses. Immunization with iOMVs increased IgG1 and IgG2c isotype levels, and the antisera from immunized mice promoted complement-dependent bactericidal and opsonophagocytic activities against *E. anophelis* C08. Our study suggests that *E. anophelis* iOMVs are a promising vaccine candidate for preventing and treating *E. anophelis* infections and provides insights into the use of OMVs in vaccine development against the clinically emergent *E. anophelis*.

**IMPORTANCE:**

*Elizabethkingia anophelis*, a Gram-negative pathogen, causes infections such as bacteraemia, pneumonia, and neonatal meningitis. The pathogen resists most antimicrobial classes, making novel approaches urgently needed. In natural settings, Gram-negative bacteria secrete outer membrane vesicles (OMVs) that carry important molecules in the bacterial life cycle. These OMVs are enriched with proteins involved in virulence, survival, and carbohydrate metabolism, making them a promising source for vaccine development against the pathogen. This study investigated the efficacy of imipenem-induced OMVs (iOMVs) as a vaccine candidate against *E. anophelis* infection in a mouse pneumonia model. Mice immunized with iOMVs were completely protected during lethal-dose challenges. Passive immunization with hyperimmune sera and splenocytes conferred protection against lethal pneumonia. Further investigation is needed to understand the mechanisms underlying the protective effects of iOMV-induced passive immunity, such as the action on specific antibody subclasses or T cell subsets.

## INTRODUCTION

The clinically emergent *Elizabethkingia* is a Gram-negative non-fermenting oxidase-, indole-, and catalase-positive rod-shaped bacteria belonging to the *Flavobacteriaceae* family of the *Bacteroidetes* phylum (1, 2). This pathogen causes bacteremia, pneumonia, and neonatal meningitis and poses great threats in clinical settings where it commonly infects immunocompromised patients and newborns (3). *Elizabethkingia anophelis* was first isolated from the midgut of the mosquito *Anopheles gambiae* in 2011 (4) and has caused several outbreaks in South Africa, the United States, Singapore, Hong Kong, and Taiwan (5). Recent investigations into the prevalence and clinical significance of *Elizabethkingia* spp. showed that *E. anophelis* accounts for a significant proportion of infections, spreading rapidly and leading to high morbidity and mortality rates (30.8%–70%) (6). Clinical *E. anophelis* isolates are resistant to most antimicrobial classes recommended for empirical treatment, including third-generation cephalosporins, β-lactams, aminoglycosides, and carbapenems (7, 8), which severely limits the treatment of *E. anophelis* infections (5). Therefore, novel approaches to counteract *E. anophelis* infections are required urgently.

In natural settings, Gram-negative bacteria secrete outer membrane vesicles (OMVs) that serve as carriers of numerous essential molecules in the bacterial life cycle (9, 10), including polysaccharides, nucleic acids, metabolites, and proteins that control biofilm formation, quorum sensing, antibiotic resistance, or virulence (11–14). Outer membrane vesiculation is modified in response to stress, particularly in harsh environments (15). Moreover, OMVs are secreted by bacteria to regulate communication and host immunomodulation (11, 16) and facilitate virulence gene transfer to the classic strain (17). OMVs are also produced with antibiotic exposure. In carbapenem-resistant *Acinetobacter baumannii*, the selective secretion of OMVs has been reported to confer carbapenem-sheltering effects (12). The findings suggest that bacteria can selectively package and release specific proteins in response to environmental challenges.

Subinhibitory concentrations of imipenem induce the release of immunologically relevant OMVs related to bacterial survival rather than promote antibiotic resistance, as observed in antibiotic stress-induced environments. In *E. anopheles*, imipenem-induced OMVs (iOMVs) are enriched with proteins involved in virulence, survival, and carbohydrate metabolism, making them a good source for vaccine development against the pathogen (18). Indeed, bacterial OMVs are a promising tool that can be employed as antigens and immunomodulators against Gram-negative bacteria (19, 20). This study investigated the efficacy of iOMVs as a vaccine candidate against *E. anophelis* infection in a mouse pneumonia model.

## RESULTS

### MIC-based antimicrobial susceptibility determination

**Fig 1:**
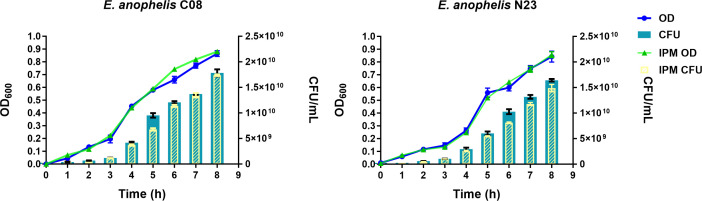
Bacterial growth curve, relative optical density (OD) values, and imipenem treatment effects. The figure shows the growth curves of *Elizabethkingia anophelis* C08 and N23 over 8 h, measured as colony-forming units (histogram) and the corresponding OD values (curve). Additionally, the effects of 1/2 MIC imipenem (4 µg/mL) treatment on the growth of the strains are presented, indicating no significant difference in growth when compared with the control.

The growth curves of *E. anophelis* strains C08 and N23 were plotted based on Optical Density at 600 nm (OD_600_) and colony-forming unit (CFU) values after incubation for 8 h at 35°C (Fig. 1). Additionally, the effects of 1/2 minimum inhibitory concentration (MIC) imipenem treatment on the growth of the strains were examined. The results indicate that there is no significant difference in growth between the control and imipenem-treated cultures for both strains. The growth curves and CFU of both strains exhibited linear correlations, with or without imipenem treatment. Broth microdilution analyses of 20 antibiotics revealed that strains C08 and N23 were not susceptible to most classes of antibiotics (Table 1). Strain C08 was susceptible to doxycycline, minocycline, and levofloxacin, while strain N23 was only susceptible to minocycline.

**TABLE 1:** Clinical antimicrobial susceptibilities of *Elizabethkingia anophelis* isolates^*a*^

Strain		C08		N23	
Antibiotic group	Antibiotic	MIC	Susceptibility^*b*^	MIC	Susceptibility^*b*^
β-Lactams	Cefotaxime	32	I	16	I
	Ticarcillin/clavulanate	>128	R	128	R
	Piperacillin/tazobactam	≥ 128	R	≥ 128	R
	Ceftazidime	>32	R	>32	R
	Cefepime	>32	R	>32	R
Tetracyclines	Tetracycline	128	R	>128	R
	Doxycycline	4	S	8	R
	Minocycline	2	S	≤ 2	S
	Tigecycline	8	R	>8	R
Polypeptides	Colistin	>4	R	>4	R
	Polymyxin B	>4	R	>4	R
Aminoglycosides	Gentamicin	>8	R	>8	R
	Amikacin	>32	R	>32	R
Fluoroquinolones	Levofloxacin	2	S	4	I
Monobactams	Aztreonam	>16	R	>16	R
Carbapenems	Imipenem/relebactam	>16	R	>16	R
	Imipenem	>8	R	>8	R
	Meropenem	>8	R	>8	R
Folate pathway antagonists	Trimethoprim/sulfamethoxazole	4	R	>4	R
	Chloramphenicol	≥ 64	R	≥ 64	R

^*a*^ MIC, minimum inhibitory concentration (μg/mL); S, susceptible; I, intermediate; R, resistant.

^*b*^ Breakpoints were adapted from Clinical and Laboratory Standards Institute (CLSI) breakpoints for other species (18).

### Virulence of *E. anophelis* strains

**Fig 2:**
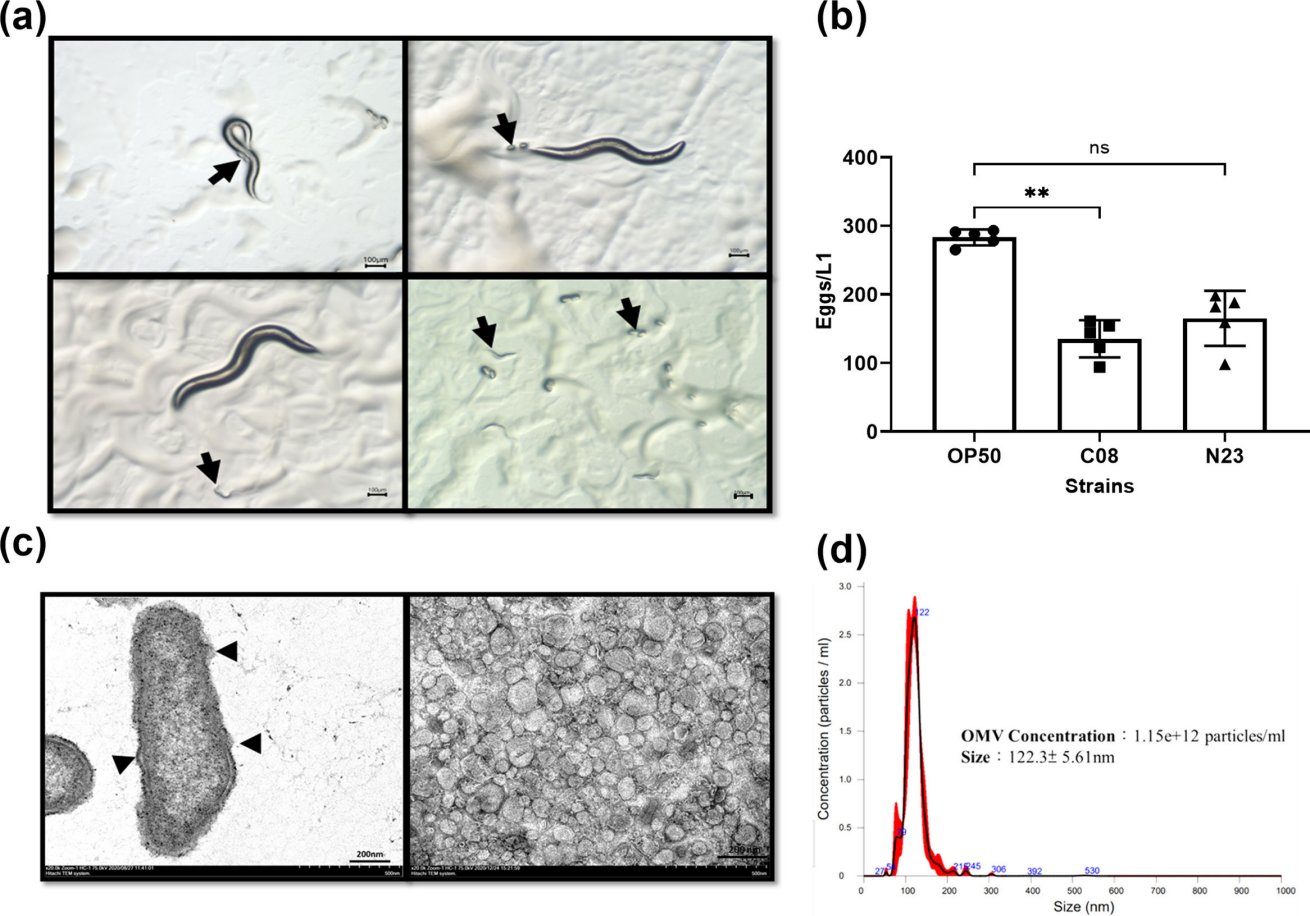
*Caenorhabditis elegans* egg count assay and physical characteristics of imipenem-induced outer membrane vesicles. (a) Representative images of *C. elegans* exposed to *E. anophelis* C08 and N23. The arrow indicates fresh eggs and L1 larva. Scale bar: 100 µm. (b) Total eggs/L1 stage worm for each strain group after 5 d. The bars represent the mean ± standard error of the mean of three independent experiments. **P < 0.01, between strain *E. anophelis* C08 and *E. coli* OP50, determined by one-way analysis of variance (ANOVA). (c) Transmission electron microscope (TEM) images of blebbing *E. anophelis* iOMVs and purified iOMVs. Scale bar: 200 nm. (d) Nanoparticle tracking analysis (NTA) of iOMVs showing a mean size of 122.3 ± 5.61 nm.

Exposure to strain C08 significantly reduced the total number of *C. elegans* eggs compared with those produced during exposure to N23 (Fig. 2a and b). These results showed the pathogenic (virulent) nature of strain C08, which was accordingly selected for further investigation.

### Purification of OMVs produced by *E. anopheli*s C08 exposed to subinhibitory concentrations of imipenem

We isolated iOMVs produced by *E. anophelis* C08 exposed to 50% of the MIC of imipenem after 6 h of incubation in Luria–Bertani (LB) broth. The morphology of the purified iOMVs was confirmed by TEM (Fig. 2c). The NTA showed that the concentration of iOMVs was 1.15 × 10^12^ particles/mL, with an average size of 122.3 ± 5.61 nm (Fig. 2d). The concentration of iOMVs increased in a dose-dependent manner with increasing concentrations of imipenem (Table S1).

### Evaluation of *E. anophelis* iOMVs as a vaccine candidate

Because no mouse model of *E. anophelis* infection is available, we established a pneumonia model by challenging the mice with four doses of *E. anophelis* (5 × 10^7^, 1 × 10^8^, 5 × 10^8^, and 1 × 10^9^ CFU). The lethal dose was 1–5 × 10^8^ (Fig. S1). Consequently, we used a challenge dose of 2 × 10^8^ CFU/mouse to assess the efficacy of the iOMVs for treating *E. anophelis*-induced pneumonia.

**Fig 3:**
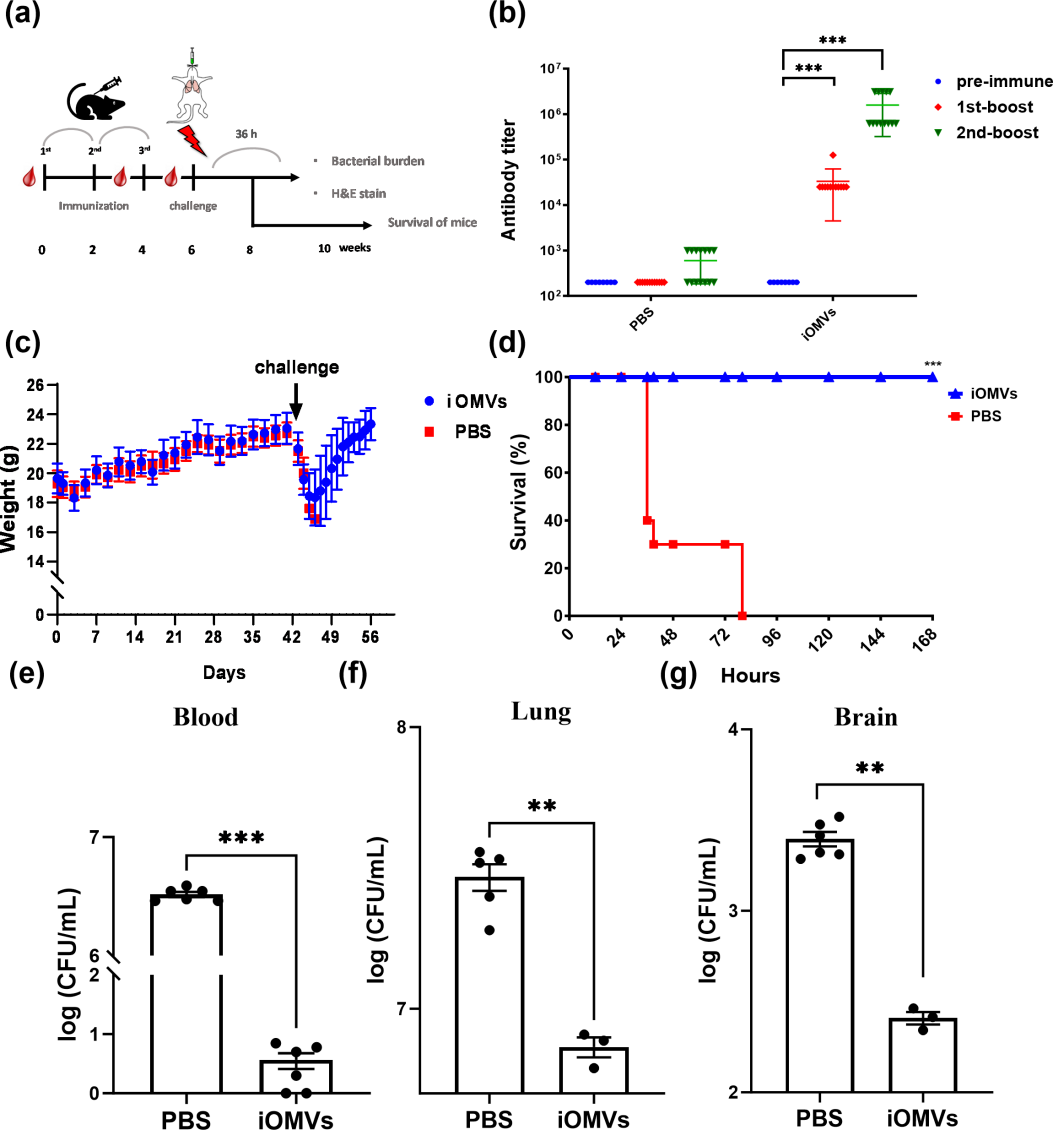
Efficacy of iOMV vaccine against *E. anophelis*-induced pneumonia. (a) Immunization schedule. (b) Total iOMV-specific IgG titer after each immunization treatment measured by enzyme-linked immunosorbent assay (ELISA). (c) Weight change during the study period, expressed as the mean ± SD. (d) Survival curves of immunized mice (*n* = 8/group) in the pneumonia model. Bacterial burden in (e) the blood, (f) lungs, and (g) brain at 36 h after infection. Data are presented as the mean CFU ± standard error of the mean (SEM) from two independent experiments with similar results; **P < 0.01 and ***P < 0.001, determined by unpaired Student’s *t*-test versus the PBS-immunized group.

Mice were immunized with three doses of iOMVs (30 µg), and each was administered 2 weeks apart (Fig. 3a). The IgG titer increased significantly after three subcutaneous immunizations (Fig. 3b). No significant changes were observed in mouse weights during the immunization period (Fig. 3c), reflecting the safety of iOMV immunization and indicating complete protection from the challenge dose (2 × 10^8^ *E. anophelis* C08; Fig. 3d). We observed a lower bacterial load in the blood, lung, spleen, and brain than that in the phosphate-buffered saline (PBS) sham group (Fig. 3e through g).

### Histopathology of the lungs, kidney, and spleen

**Fig 4:**
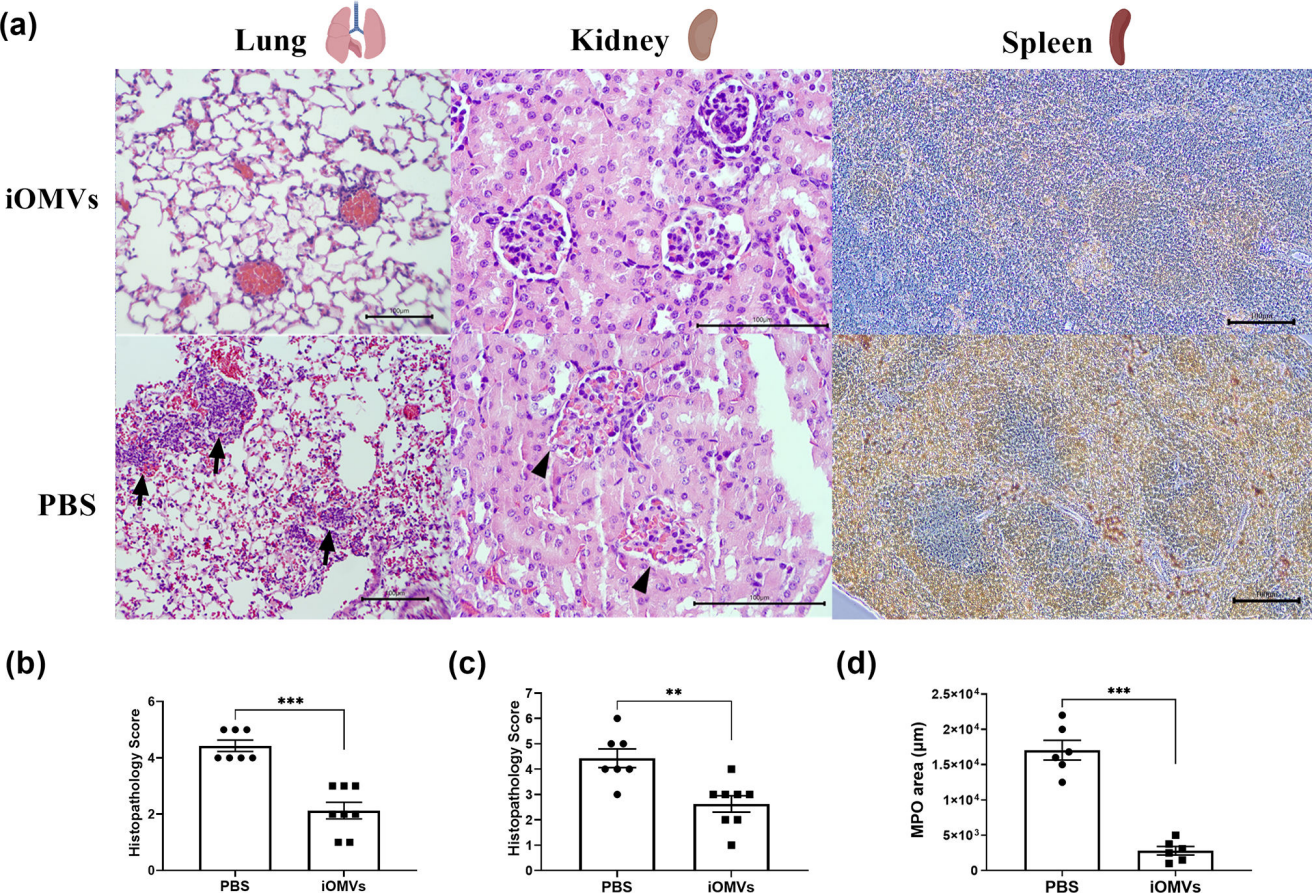
Histopathology of the lungs, kidney, and spleen in *E. anophelis*-infected mice after iOMV or PBS immunization. (a) HE staining of lung and kidney tissues from mice immunized with iOMVs or PBS at 36 h after infection. Images were taken at 40× magnification using light microscopy; representative sections from three mice per group are shown. The arrows indicate regions of perivascular and peribronchial infiltration, and the arrowhead indicates renal tubules and glomeruli with swollen pale epithelial cells with some pyknotic or karyorrhectic nuclei (reflecting degeneration and necrosis, respectively). MPO staining of the spleen shows polymorphonuclear cell-specific MPO production. Scale bar = 100 µm. Histopathology severity scores for (b) the lungs, (c) kidney, and (d) spleen of infected mice immunized with iOMVs or PBS. Data are presented as the mean ± SEM (*n* = 6/group) of two independent experiments; **P < 0.01 and ***P < 0.001, determined by unpaired Student’s *t*-test versus the PBS control group.

The histological analysis showed more inflammatory infiltration in the lungs, kidney, and spleen of the PBS control mice than in the immunized mice (Fig. 4a). Hematoxylin and eosin (HE) staining was performed on the lung and kidney sections from iOMV- and PBS-immunized mice infected with *E. anophelis* C08. The PBS group exhibited neutrophil infiltration in the peri-bronchial vasculature of the lung tissue, diminished, and distorted glomeruli, leukocyte infiltration, and edema exudate in the kidney tissue. In contrast, tissues from the iOMV-immunized group had a healthy structure and low histopathology score (Fig. 4b and c). To further evaluate the immune response, we performed myeloperoxidase (MPO)-based immunohistochemical analyses on the spleen sections. The PBS group exhibited ill-defined spleen sections with distorted lymphoid architecture, diffuse white pulp, and giant macrophages, whereas the iOMV-immunized group had well-defined spleen sections with healthy lymphoid follicles and sinuses and showed a significantly reduced MPO-stained area compared with that in the PBS group (Fig. 4d).

### *Ex vivo* immune response elicited by iOMVs

**Fig 5:**
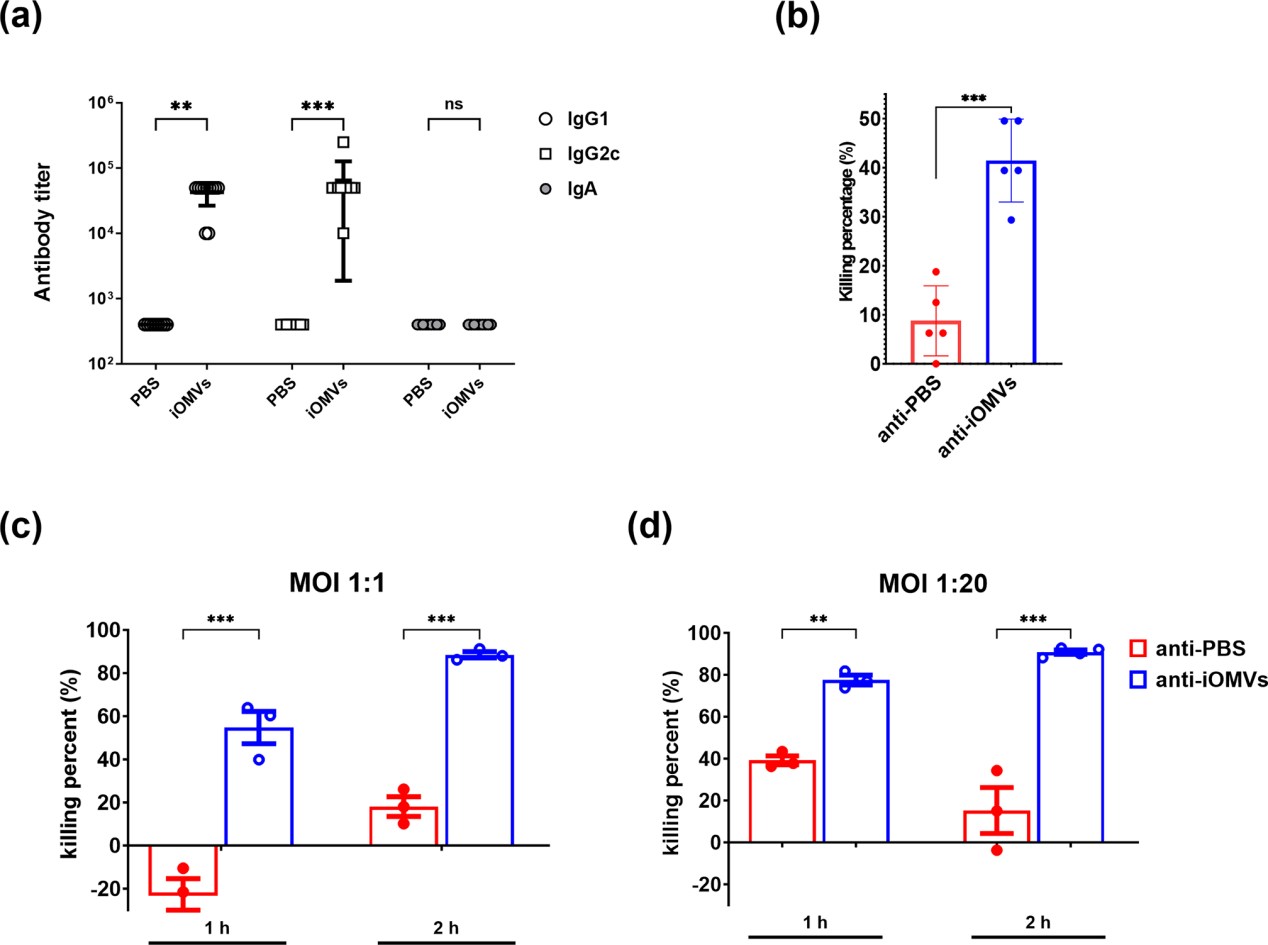
Isotype analysis of IgG antibody and bactericidal activity induced by iOMV vaccine. (a) Distribution of IgG subclasses (IgG1 and IgG2c) after three rounds of immunization with *E. anophelis* iOMVs. (b) Complement-dependent bactericidal activity of antisera to iOMVs of *E. anophelis* C08 in the presence of human complement molecules for 1 h. (c and d) Macrophage-mediated bacterial killing percentage at a 1:1 and 1:20 multiplicity of infection (MOI) for 2 h. The bactericidal activity of the antisera was calculated as the difference in inhibition/killing activity compared with that of PBS-immunized control antisera. Data are presented as the mean CFU ± SEM from two independent experiments with similar results; **P < 0.01 and ***P < 0.001, determined by unpaired Student’s *t*-test versus the PBS-immunized group.

We investigated the protective mechanism of the *E. anophelis* iOMV vaccine by examining the iOMV-specific IgG isotype in the sera of immunized mice. The expression of IgG subclasses is influenced by several factors, including the prevailing cytokine environment (21). Particularly, interferon (IFN)-γ (a Th1 cytokine) and interleukin (IL)-4 (a Th2 cytokine) induce isotype switching to IgG2c and IgG1, respectively (21). The results showed that IgG1 and IgG2c isotypes were dominant, indicating the induction of Th2 and Th1 immune responses, respectively (Fig. 5a). Notably, mice vaccinated with iOMVs subcutaneously did not exhibit a detectable iOMV-specific IgA response.

We evaluated the humoral immune response generated by iOMV immunization using serum bactericidal and opsonophagocytosis assays. The bactericidal assay showed that the antisera from iOMV-immunized mice promoted a complement-dependent bactericidal effect on *E. anophelis* C08, unlike that from the control mice (*P* < 0.01; Fig. 5b). Additionally, the opsonophagocytosis assay showed that bacterial growth was significantly inhibited by sera from iOMV-immunized mice at different time points (1 and 2 h) and MOIs, unlike that from the PBS control mice (Fig. 5c and d). These findings suggest that iOMV immunization induced specific humoral immune responses, which contributed to the enhancement of bacterial clearance through complement-dependent bactericidal and opsonophagocytic activities.

**Fig 6:**
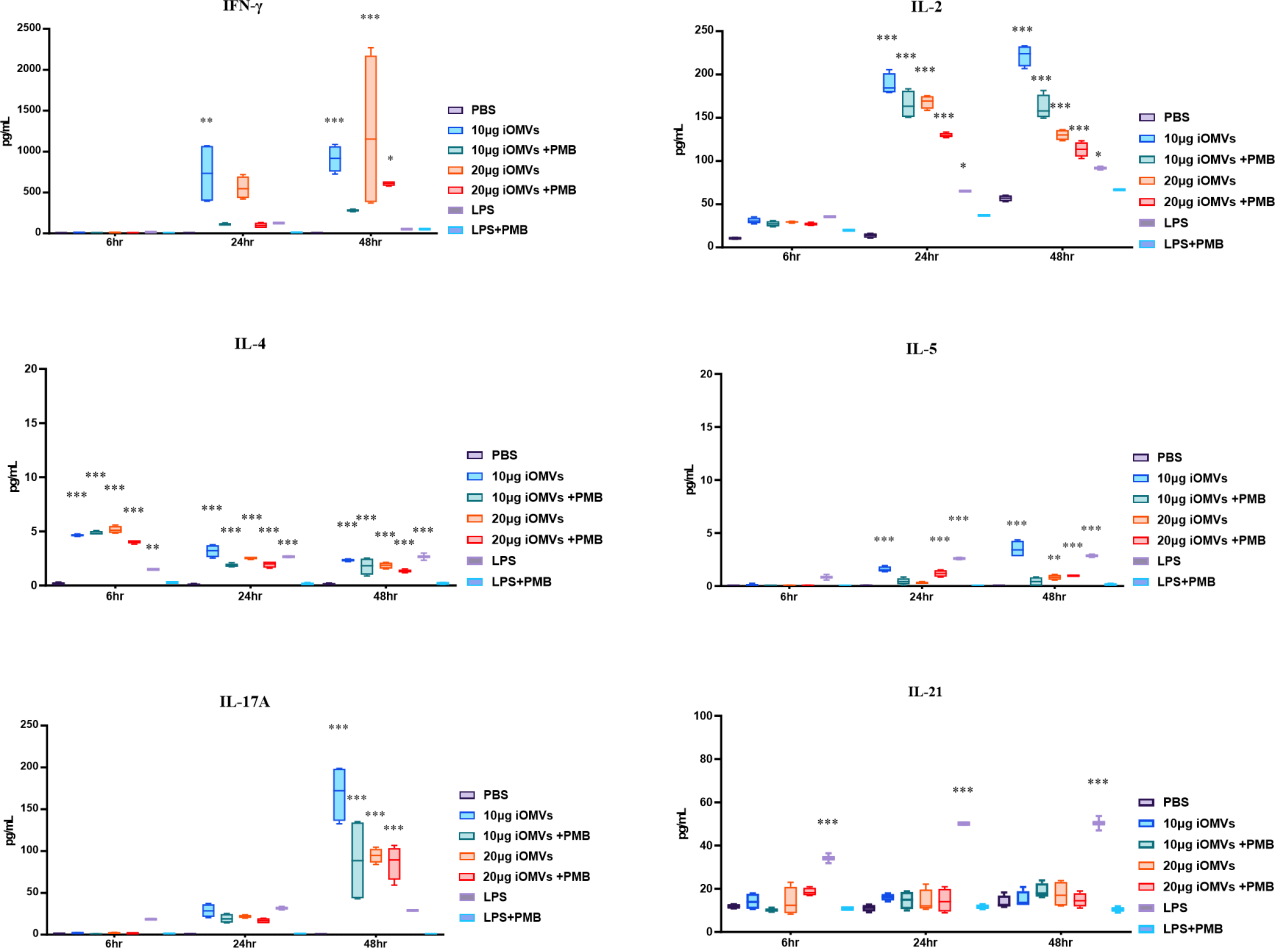
Comprehensive analysis of iOMV-specific cellular immune responses. CD3^+^ cells were isolated from mouse spleens on day 42 postsecond immunization and cultured for 6, 24, and 48 h. The cells were then stimulated with varying concentrations of iOMVs (10–20 µg/mL), LPS, and PBS, both with and without PMB. Cytokine levels of IFN-γ, IL-2, IL-4, IL-5, IL-17A, and IL-21 were quantified using commercial ELISA kits. Data are presented as mean ± SEM. Statistical significance was determined using a two-way ANOVA, followed by Bonferroni’s multiple comparison test, to adjust for multiple comparisons and control the family-wise error rate. Asterisks denote levels of significance: *P < 0.05, **P < 0.01, and ***P < 0.001.

To further explore the immune response elicited by iOMVs, splenocytes and CD3^+^ T cells were harvested from immunized mice and cultured *ex vivo* with different concentrations of the vaccine antigen. Notably, splenocytes from iOMV-immunized mice secreted a significantly higher amount of IFN-γ than those from the PBS mice (Fig. S2). We assessed cytokine production by the CD3^+^ T cells at 6, 24, and 48 h postinfection (hpi) with 10 and 20 µg of iOMVs and observed a significant upregulation in the levels of key proinflammatory cytokines, including IFN-γ, IL-2, IL-4, IL-5, and IL-17A, whereas the levels of IL-21 did not change significantly over time (Fig. 6). Polymyxin B (PMB) was added to all samples to neutralize the influence of lipopolysaccharides (LPSs), and the concentrations of IL-2, IL-4, and IL-17A in iOMV-treated samples remained significantly higher than those in the PBS control group samples. The results highlight the adjuvant role and indicate that factors other than LPSs within the iOMV vaccine are involved in inducing an effective immune response, influence the ability of OMVs to elicit robust cellular and humoral immune responses, and modulate the immune response by OMVs (22).

### Passive immune effects

**Fig 7:**
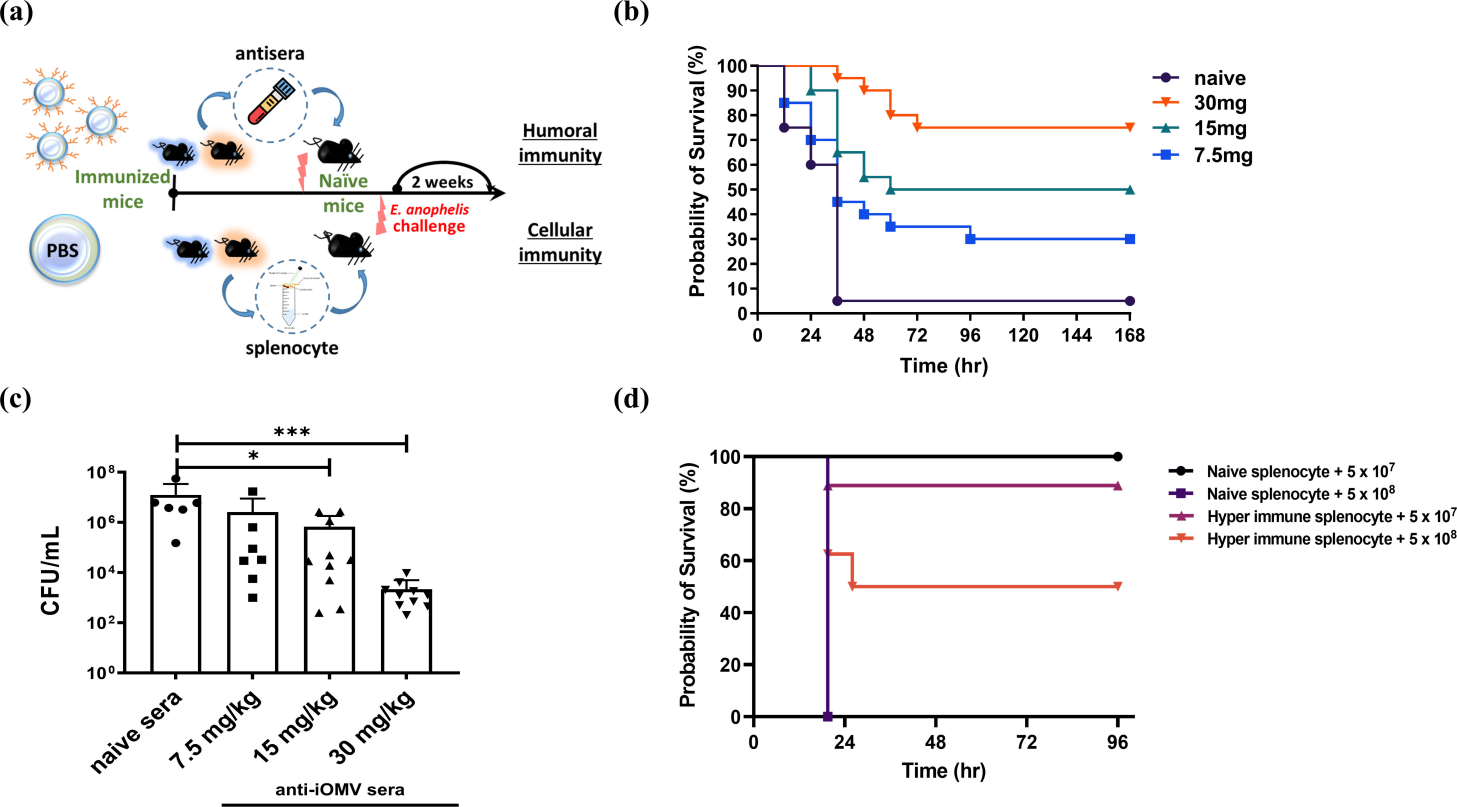
Humoral and cellular immune responses in the mouse pneumonia model of passive immunity. (a) Schematic representation of the mouse model for passive immune induction. (b) Probability of survival with dose-dependent humoral immune response after a lethal dose of *E. anophelis* C08. (c) Bacterial burden 36 hpi under different concentrations of anti-iOMV sera. (d) Probability of survival with a cellular immune response after infection with 5 × 10^7^ and 5 × 10^8^ *E. anophelis* C08. Data are presented as the mean ± SEM of two independent experiments with similar results; *P < 0.01 and ***P < 0.001, determined by a log-rank test and one-way ANOVA versus the control group.

In addition to active immunization, we evaluated the potential of iOMV-specific antisera and hyperimmune splenocytes to confer passive immunity against *E. anophelis*-induced pneumonia. We found that the hyperimmune sera had a dose-dependent protective effect (Fig. 7a and b) and reduced the bacterial burden (Fig. 7c). Similarly, after inoculation of hyperimmune splenocytes, the mice showed a 50% higher survival rate than mice inoculated with naive splenocytes (Fig. 7d).

## DISCUSSION

The emergence of *E. anophelis* outbreaks and the associated morbidity and mortality rates highlight the urgent need for effective treatment strategies (5). The efficacy of OMVs as vaccine candidates has been demonstrated against various bacterial pathogens. For instance, OMVs from *Neisseria meningitidis* (23), *Shigella* species (24), *A. baumannii* (25), and *Vibrio cholerae* (26) are immunogenic, possess self-adjuvant properties, and are endocytosed by immune cells to confer protection against bacterial infection in animal models (27). Furthermore, OMVs are safe and well tolerated in clinical trials and can be engineered to express heterologous antigens, making them an attractive platform for vaccines with cross-protection (23, 28).

Even though *E. anophelis* is generally resistant to most antibiotics, virulence differs among strains or could even be strain specific (29). The *C. elegans* egg count assay—a reliable model for evaluating the pathogenicity of bacterial strains that has been extensively used in research on host-parasite interactions and innate immunity—was used to identify virulent strains from clinical isolates (30). Gardner et al. developed an egg-laying assay to measure the effects of bacteria on *C. elegans* behavior (31). They observed that egg-laying behavior is modulated by neurotransmitters, such as serotonin and acetylcholine, and requires the coordinated activity of several neurons and muscle groups. Similarly, *Elizabethkingia* spp. prefer to be parasitic in nerve tissue, causing significant neuronal necroinflammation in the brain and spine (32). In addition, the reduction in *C. elegans* egg production may reflect a protective response to unfavorable environmental conditions (33). The significant reduction in egg production observed in our study after exposure to *E. anophelis* C08 suggested that this strain possessed virulence factors that impact host reproductive fitness (31, 34). This finding was consistent with results from our previous study indicating that iOMVs support the production of virulence factors and survival-related proteins, reflecting their potential for vaccine development (18).

Because they resemble the bacterial antigenic surface of the pathogen, OMVs, which naturally contain a complex mixture of antigens, such as outer membrane proteins, LPSs, and bacterial components, have been shown to stimulate humoral and cell-mediated immune responses (22). In our study, subcutaneous immunization with *E. anophelis* OMVs induced an outstanding humoral immune response, as evidenced by high levels of OMV-specific IgG1 and IgG2c antibodies, effectively protecting mice from a lethal challenge with *E. anophelis*. The comparable titers of IgG1 and IgG2a generated a balanced Th1/Th2 humoral response, which was consistent with previous reports indicating that OMVs promoted dendritic cell maturation (35). Additionally, we demonstrated that iOMV immunization could induce a long-lasting antibody response, with detectable antibody titers maintained for up to 12 months after the final immunization (Fig. S3). A significant increase in the production of crucial proinflammatory cytokines, including IFN-γ, IL-2, IL-4, IL-5, and IL-17A, was also observed in iOMV-treated splenocytes, suggesting that the vesicles elicit a robust immune response that is essential in protecting against bacterial infection (20) and that the responses of Th1 and Th17 are critical for the clearance of bacterial pathogens (36). Therefore, the induction of these specific cytokines by the iOMV vaccine may indicate that enhanced Th cell differentiation may elicit a protective immune response against *E. anophelis* infection.

Finally, passive immunization with iOMVs may be a promising approach for the prevention and treatment of bacterial infections. The passive transfer of extracellular vesicle (EV)-specific serum and splenocytes conferred protection against *K. pneumoniae* infection (37). Specifically, the EV-specific serum provided protection by neutralizing *K. pneumoniae* virulence factors, while EV-specific splenocytes mediated protection by producing cytokines and activating macrophages and neutrophils to clear *K. pneumoniae* from infected tissues. In the current study, iOMVs provided active and passive immunizations through iOMV-specific antisera and hyperimmune splenocytes that partially protected against lethal *E. anophelis*-induced pneumonia *in vivo*.

This is the first study elucidating the application of OMVs as a vaccine against *E. anophelis*. The protective and immunogenic effects of *E. anophelis* iOMVs indicated complete protection in the *E. anophelis-*induced pneumonia mouse model. The bacterial loads and histopathology of infected organs were also less severe in immunized mice than those of untreated mice. Immunization with iOMVs increased IgG2c and IgG1 isotype levels when compared with the levels in the PBS control, indicating robust activation of both Th1 and Th2 immune responses, without a clear skew toward either. The balanced humoral response makes iOMVs a promising *E. anophelis* vaccine candidate. The antisera from immunized mice promoted complement-dependent bactericidal and opsonophagocytic activities against *E. anophelis*. Additionally, passive immunization with hyperimmune sera and splenocytes conferred protection against lethal pneumonia. Our results provide valuable insights and a promising approach to combat the clinically troublesome *E. anophelis* using OMVs as a vaccine candidate.

Despite the promising prospects of using iOMVs, several challenges remain in the development of effective vaccines against bacterial infections (38). For example, OMVs can contain protective and non-protective antigens, making it essential to identify and prioritize antigens that induce protective immune responses (20, 39). OMV-based vaccines have shown limited efficacy against diverse bacterial strains, which may require the inclusion of multiple OMVs or heterologous antigens to achieve broad-spectrum protection (40). Additionally, the stability and scalability of OMV-based vaccines need to be further optimized for their practical use (41).

In conclusion, our study demonstrates the potential of *E. anophelis* iOMVs in vaccine development against the infections caused by this emerging pathogen and the efficacy of immunity induced by OMV-specific antisera. Further studies are needed to optimize the use of OMVs as a component of passive immunization and identify optimal dosages, administration routes, and protective components for achieving maximum efficacy (42). The mechanisms underlying the protective effects of iOMV-induced passive immunity, such as the action on specific antibody subclasses or T cell subsets, require further investigation.

## MATERIALS AND METHODS

### Bacterial strain, growth conditions, and antimicrobial treatment

*E. anophelis* strain C08 (NCBI accession number: CP104875) and N23, clinically isolated from blood cultures of female patients aged 73 and 18 years, respectively, were obtained from the Microbial Infections Reference Laboratory of the National Health Research Institutes (Taipei, Taiwan). These strains were cultured aerobically overnight in LB broth or agar at 35°C with shaking at 250 rpm and passaged to the mid-log growth phase in LB at 35°C with shaking at 250 rpm for 4 h. Subcultures were rinsed three times with PBS and resuspended in PBS. For all *in vivo* experiments, the inoculum was delivered prior to treatment.

### Broth microdilution assay

Broth microdilution was conducted in accordance with the Clinical and Laboratory Standards Institute (CLSI) guidelines (43). We diluted 0.5 McFarland inoculum (100 µL) to 1:100 in 10 mL cation-adjusted Mueller Hinton broth (CAMHB; BD BBLTM, Thermo Fisher Scientific, Merelbeke, Belgium) and transferred 50 µL of diluted inoculum to all wells of a 96-well plate containing 50 µL of CAMHB with or without an antibiotic (1:2 dilution), thus producing an inoculum of ±5 × 10^5^ CFU/mL. The 96-well plates were tightly sealed with adhesive foil and stored in an incubator for approximately 24 h at 35°C. Strains were designated as susceptible (S), intermediate (I), and resistant (R) based on their respective minimal inhibitory concentration values and the CLSI-defined interpretive criteria.

### *Caenorhabditis elegans* egg count assay

The *C. elegans* egg count assay was performed to determine bacterial virulence as previously described (31). Briefly, one L4 stage worm was placed on lawns made of *Escherichia coli* OP50 or *E. anophelis* C08 or N23 grown on nematode growth medium (NGM) agar plates. Over 5 d, worms were transferred daily onto freshly prepared plates. Three plates were examined for the presence of eggs/L1 stage worms. The egg production of nine worms was assayed for all strains of bacteria tested.

### Isolation and purification of iOMVs

Briefly, to isolate iOMVs, *E. anophelis* subcultures were grown for 6 h in 4 µg/mL imipenem in LB broth until the log phase (OD_600_ = ~0.5) (18). The culture was pelleted by centrifugation at 6,000 × *g* and 4°C for 20 min. The supernatant was filtered using 0.22 µm Polyethersulfone (PES) membrane filters. The filtrate was concentrated using the continuous diafiltration mode of a Minimate TFF System and a Capsule with an Omega 100K Membrane (Pall Corporation, Ann Arbor, MI, USA). The flow through was harvested by size-exclusion chromatography and subjected to ultracentrifugation at 150,000 × *g* for 3 h at 4°C to pellet the OMVs. The purified OMV pellets were washed with PBS and carefully resuspended in 1000 µL PBS. The vesicle suspension was checked for bacterial contamination by plating in LB agar, and the purified suspensions were stored at 4°C for use within 2 weeks or −80°C for future analysis.

### Transmission electron microscopy

For TEM, the bacterial pellets obtained during OMV isolation were fixed in 2% paraformaldehyde and 2.5% glutaraldehyde in PBS and mixed thoroughly. Fixed samples were washed in 0.1 M sodium cacodylate (pH 7.24), postfixed with buffered 2% OsO_4_, water washed, and dehydrated in a graded ethanol series (25%–100%) with 25% increments. The samples were then infiltrated in a graded series of Epon resin (Ted Pella, Redding, CA, USA) for 2 d, embedded in fresh Epon resin, and polymerized at 60°C for 48 h. Purified OMV pellets were resuspended in 2% paraformaldehyde and floated onto a 200-mesh carbon-coated Formvar nickel grid (EMS, Hsin An Instruments, Taiwan) for 5 min. Excess solution was removed with filter paper, and the sample grid was floated on a 10-µL droplet of 1% aqueous uranyl acetate for 30 s. The stain was removed with filter paper, and the sample was air dried and subsequently imaged using a Hitachi HT7700 transmission electron microscope (Hitachi, Tokyo, Japan).

### Nanoparticle tracking analysis

Nanoparticle size distribution was determined using a NanoSight NS300 system (Malvern Panalytical, Malvern, UK). Samples were diluted 100 times in 1 mL PBS. Diluted OMVs were measured based on Brownian movement and loaded onto the NanoSight NS300, and particle size was recorded for 60 s per technical replicate. Five technical and three biological replicates were performed per sample. NTA results reflected the particle number per mL of OMV solution. The protein concentration of each sample was quantified using a bicinchoninic acid (BCA) assay (Thermo Fisher Scientific, Waltham, MA, USA).

### Mouse sepsis and pneumonia models

All animal studies were approved by the National Defense Medical Center Institutional Animal Care and Use Committee (NDMC IACUC-21–037). Female C57BL/6 mice (10 weeks old) were provided by the National Laboratory Animal Center (Taipei, Taiwan) and bred in a barrier facility under specific pathogen-free conditions. The mice (*n* = 8/group) were grouped for administration of four challenge doses (5 × 10^7^, 1 × 10^8^, 5 × 10^8^, and 1 × 10^9^) via intratracheal and intraperitoneal routes. Survival rates and weights were recorded for 14 d to determine the lethal dose.

### Immunization and hyperimmune sera generation

For 15 7- to 8-week-old female C57BL/6 mice per group, blood was collected 2 d before the start of the immunization schedule and pooled to serve as the negative control in all the dose experiments. All mice received 30 µg of iOMVs subcutaneously, followed by two booster doses at 14-d intervals with the same quantity of protein.

### Active and passive immunity mouse models

For the active immunity model, C57BL/6 mice (*n* = 10/group) were subcutaneously immunized with 30 µg of iOMVs on days 0, 14, and 28. Blood samples were collected before the last immunization and tested against each immunogen. IgG antibody titers were determined using an antigen-specific enzyme-linked immunosorbent assay. We established an oropharyngeal model of aspiration pneumonia that recapitulates hospital- or ventilator-associated pneumonia (relevant to intensive care unit populations) (44).

For the passive immunity model, naive C57BL/6 mice (*n* = 10/group) were injected with either sera or splenocytes (hyperimmune anti-OMV or PBS-immunized) and challenged with a lethal dose of *E. anophelis* C08. To induce humoral immunity, we immunized mice with either PBS or iOMVs to produce antisera, which was then injected into mice at different dosages (7.5, 15, and 30 mg/kg) 2 h postinfection (hpi) with a lethal dose of *E. anophelis* C08 (Fig. 7a). To induce cellular immunity, we immunized mice with either PBS or iOMVs to produce naive or hyperimmune splenocytes. The splenocytes purified from iOMV-immunized mice were inoculated intraperitoneally into mice (10^6^/mouse) 24 h before the *E. anophelis* challenge.

### Histopathological analysis of immunized mice

The immunized mice were challenged intratracheally on day 42 with a lethal dose of 3 × 10^8^ CFU, mixed with 10% porcine mucin (type 3; Sigma-Aldrich, Taiwan) of mid-log phase *E. anophelis* C08. At 36 hpi (when the control mice were expected to begin dying), organs were harvested and homogenized in sterile PBS. The homogenized organs were quantitatively cultured for each separate mouse to determine the bacterial burden. The excised organs were placed in vials containing 4% formaldehyde and stained with hematoxylin and eosin and myeloperoxidase antibody (ab188211, Abcam, Cambridge, UK) for immunohistochemical analysis of neutrophilic granulocytes. Multiple tissue sections of each organ were scored by a blinded pathologist for injury severity, as previously described (45, 46). MPO-positive areas were imaged using a Nikon 90i Eclipse widefield microscope (Nikon Instruments Inc., Melville, NY, USA) and quantified using NIS-Elements software.

### ELISA

ELISA was performed to evaluate the antigenicity of OMVs as previously described (46). Briefly, plate wells were coated overnight with 50 ng of proteins and blocked with 5% skim milk (wt/vol) in PBS. Mouse antisera were serially diluted fivefold with PBS containing 1% bovine serum albumin (BSA; Sigma-Aldrich, St. Louis, MO, USA) and added to the wells. We then added horseradish peroxidase-conjugated anti-mouse IgG (KPL, Gaithersburg, MD, USA), IgG2a-, IgG2c-, and IgA-specific antibodies diluted in PBS containing 1% BSA and TMB peroxidase substrate (KPL). Optical density at 450 nm absorbance was measured to determine specific antibody titers on a SpectraMax M5 system (Molecular Devices, Sunnyvale, CA, USA). IgG1 and IgG2a antibody responses were evaluated by ELISA using specific anti-mouse isotype antibodies.

### Cytokine analysis

Culture supernatants were collected at each time point and stored at –80°C until further analysis. We determined the concentrations of interferon (IFN)-γ, interleukin (IL)−2, IL-4, IL-5, IL-17A, and IL-21 in the supernatants using a commercially available ELISA kit according to the manufacturer’s instructions. The OD was measured at 450 nm using the microplate reader, and cytokine concentrations were calculated based on the standard curve generated with recombinant cytokines.

### Complement-dependent bactericidal assay

The complement source (human sera) was absorbed three times for 30 min at 4°C with the target *E. anophelis* C08 to remove non-specific antibodies. *E. anophelis* C08 was grown until a final concentration of 10^6^ CFU/mL. In the wells of sterile flat-bottom microtiter plates, 10 µL of heat-inactivated antisera (immunized mouse sera at dilutions of 1:10) was mixed with 10 µL of the bacterial suspension (10^4^ cells/well) and 80 µL of undiluted human complement. The microtiter plates were incubated for 1 h at 37°C with shaking (80 rpm), after which the samples were diluted and plated for bacterial enumeration. The bactericidal activity of the antisera was calculated as the difference in survival rates between the immunized and PBS–control antiserum treatments.

### Opsonophagocytosis assay

The opsonophagocytosis assay was conducted as previously described (18). Briefly, J774A.1 murine macrophages (ATCC CCL-240) were cultured in Dulbecco’s modified Eagle’s medium (DMEM), GeneDireX supplemented with 10% heat-inactivated fetal bovine serum (FBS; Gibco), and 100 U/mL penicillin-streptomycin (GeneDireX). Cells were incubated at 37°C with 5% CO_2_. The bacterial pellet was washed twice with PBS and resuspended in DMEM before the assays. Reactions were initiated by incubating 1 × 10^5^ or 2 × 10^5^ CFU (multiplicity of infection = 1:1 or 1:20) of *E. anophelis* C08 with heat-inactivated test serum and iOMVs in the wells. After a 2-h incubation with gentle shaking (80 rpm), the samples were serially diluted and plated. Bacterial killing rates were determined by comparing the difference in CFU between the iOMV-immunized serum and PBS-immunized antiserum treatments. Hyperimmune serum from OMV-immunized mice was used as the positive control.

### CD3^+^ T cell isolation and OMV stimulation assay

For splenocyte isolation, the immunized mice were euthanized and their spleens were aseptically removed and placed in ice-cold RPMI 1640 medium supplemented with 10% FBS, 100 U/mL penicillin, and 100 µg/mL streptomycin. Spleens were gently dissociated using the frosted ends of two sterile microscope slides, and the cell suspension was passed through a 70-µm cell strainer. The cells were collected by centrifugation at 500 × *g* and 4°C for 5 min, and red blood cells were lysed using ammonium–chloride–potassium (ACK) lysis buffer. Splenocytes were washed twice with PBS and resuspended in complete RPMI 1640 medium.

Purification of CD3^+^ cells was performed using an EasySep Mouse CD3^+^ T Cell Isolation Kit (STEMCELL Technologies) according to the manufacturer’s instructions. Briefly, single-cell suspensions of splenocytes were prepared and incubated with the kit’s antibody cocktail, followed by treatment with magnetic nanoparticles conjugated to anti-biotin antibodies. CD3^+^ cells were negatively selected by magnetic separation, yielding a purity of >95%. Purified CD3^+^ cells were stimulated with iOMVs at concentrations of 10 and 20 µg/mL, along with polymyxin B (10 µg/mL) to neutralize the effects of LPSs, for 6, 24, and 48 h in complete RPMI 1640 medium supplemented with 10% FBS and 50 µM β-mercaptoethanol.

### Statistical analysis

All assays were performed in triplicate, and the mean ± standard deviation was calculated. GraphPad Prism 9.0 (GraphPad Software, San Diego, CA, USA) was used to evaluate the differences between groups, with statistical significance set at *P* < 0.05. Survival rates were compared using a non-parametric log-rank test. A two-way analysis of variance test with Bonferroni’s multiple comparison was used to investigate the interaction effects of two independent variables—immunization and time—on cytokine levels. The approach allows for a more comprehensive understanding of the complex relationships among our data while controlling for Type I errors. Graphics were created with BioRender.com (accessed on 2 May 2023).

## Data Availability

All data generated or analyzed during this study are included in the published article and its supplementary materials.

## SUPPLEMENTAL MATERIAL

Supplemental figures and table msphere.00400-23-s0001.docx Fig. S1-S3, Table S1, and graphical abstract.

ASM does not own the copyrights to Supplemental Material that may be linked to, or accessed through, an article. The authors have granted ASM a non-exclusive, world-wide license to publish the Supplemental Material files. Please contact the corresponding author directly for reuse.
