# Supplementary material for: Elizabethkingia anophelis outer membrane vesicles as a novel vaccine candidate against infection: insights into immune response and potential for passive immunity
Source: mSphere. 2023 Nov 28;8(6):e00400-23. doi: 10.1128/msphere.00400-23 (PMC10732079; doi:10.1128/msphere.00400-23)

# Supplemental Material

Table S1. Physical characteristics of *Elizabethkingia anophelis* C08 outer membrane vesicles under different imipenem concentrations.

| Antibiotic^a^ | Protein (μg/mL) | iOMV concentration  (Particles/mL) | CFU/mL | Particles/CFU | Particle size (nm) |
| --- | --- | --- | --- | --- | --- |
| Control | 19.24 | 1.36 × 10^11^ | 1.86 × 10^10^ | 7.33 | 115.3 |
| IMP × 1/4 | 30.74 | 6.99 × 10^11^ | 2.14 × 10^10^ | 32.74 | 117.5 |
| IMP × 1/2 | 50.45 | 1.15 × 10^12^ | 1.59 × 10^10^ | 71.81 | 113.7 |
| IMP × 1 | 52.09 | 1.50 × 10^12^ | 2.12 × 10^10^ | 70.84 | 119.5 |

^a^Minimal inhibitory concentrations of imipenem (IMP) were used: × 1 = 8 mg/mL, × 1/2 = 4 mg/mL, and × 1/4 = 2 mg/mL.

CFU, colony forming unit; iOMV, imipenem-induced outer membrane vesicle.


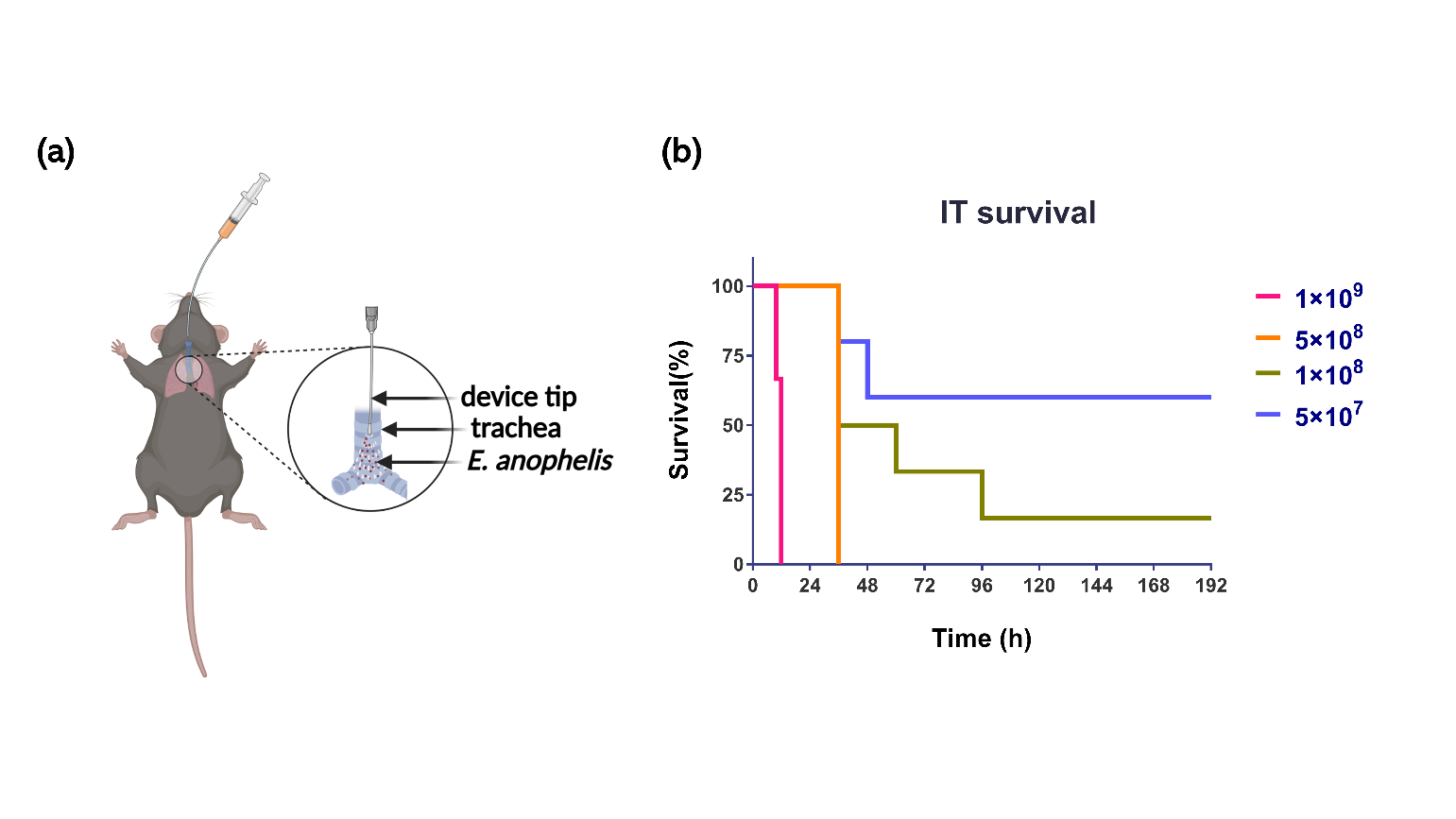


Figure S1. Mouse pneumonia model of *Elizabethkingia anophelis* infection.

(a) Schematic representation of intratracheal inoculation. Mice were anesthetized with an intraperitoneal injection of xylazine and ketamine, followed by intratracheal inoculation with an appropriate number of *E. anophelis* C08 in 20 μL saline solution. The actual inoculum in each experiment were determined by plating 10-fold serial dilutions on brain heart infusion agar plates. (b) Survival curves of mice inoculated with increasing amounts of bacterial colony forming units (CFUs). Survival results are expressed as percentages from two independent experiments (n = 8/group).


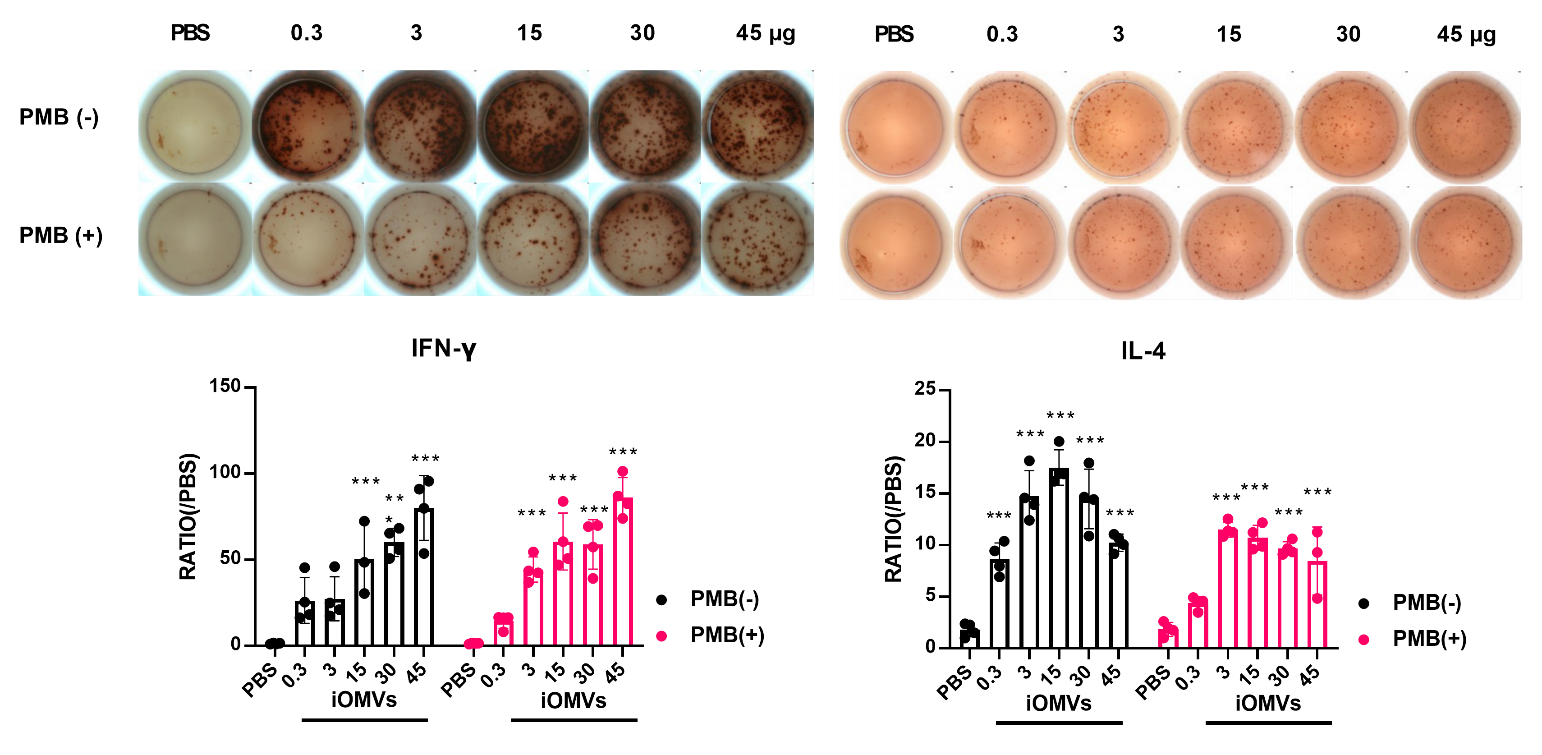


Figure S2. Analysis of IFN-γ and IL-4 levels by ELISPOT assays.

Single-cell suspensions were prepared from the spleens of mice on day 42 post-second booster immunization. Splenic lymphocytes were cultured for 24 h and stimulated with varying concentrations of imipenem-induced outer membrane vesicles (iOMVs) (0, 0.3, 3, 15, 30, and 45 μg), either with or without polymyxin B (PMB). IFN-γ and IL-4 secretion levels were quantified using ELISPOT assays, performed in triplicate wells. The data represent two independent experiments yielding similar results. Statistical analysis was conducted using a two-way ANOVA with Bonferroni’s multiple comparison test, to evaluate the effects of iOMV concentration and PMB presence on cytokine secretion. Data are presented as mean ± SEM; **P <0.01, ***P <0.001, compared to the PBS-immunized control group.

Figure S3. Long-lasting iOMV-specific antibody response compared to that of the PBS-immunized group.

Antibody titers specific to imipenem-induced outer membrane vesicles (iOMVs) were analyzed by ELISA at 1, 2, 3, 6, 9, and 12 months post-immunization. Data for a PBS-immunized control group have been included for reference. The antibody titers in the iOMVs-immunized group were consistently higher than those in the PBS-immunized group at multiple time points, confirming a long-lasting and robust iOMV-specific antibody response. Statistical significance was determined using one-way ANOVA with Dunnett’s multiple comparison test; asterisks indicate levels of significance compared to that of the PBS-immunized group (*P <0.05, **P <0.01, ***P <0.001).

**Graphic Abstract**


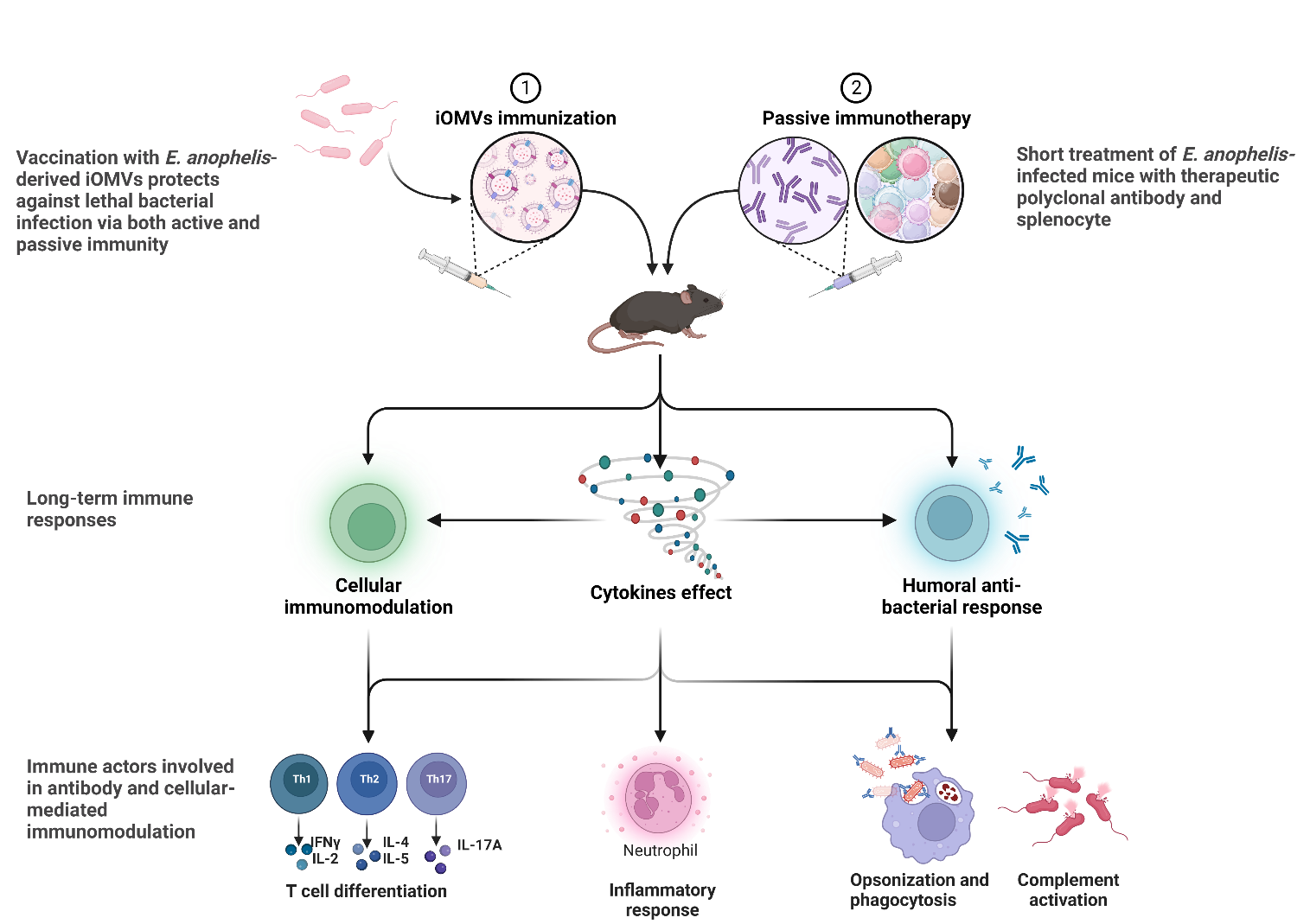

Supplement: Supplemental figures and table — Fig. S1-S3, Table S1, and graphical abstract. [file msphere.00400-23-s0001.docx]
